# Stakeholder’s Perceptions of Mexico’s Federal Corn Flour Fortification Program: A Qualitative Study

**DOI:** 10.3390/nu12020433

**Published:** 2020-02-08

**Authors:** Anna W. Waller, Astrid Dominguez-Uscanga, Emely Lopez Barrera, Juan E. Andrade, Jeanette M. Andrade

**Affiliations:** 1 Department of Food Science and Human Nutrition, University of Illinois at Urbana-Champaign, Urbana, IL 61801, USA; awaller2@illinois.edu (A.W.W.); astrid86@illinois.edu (A.D.-U.); eclb@illinois.edu (E.L.B.); jandrade@illinois.edu (J.E.A.); 2 Division of Nutritional Sciences, University of Illinois at Urbana-Champaign, 572 Newell Dr., Urbana, IL 61801, USA; 3 Food Science and Human Nutrition Department, University of Florida, Gainesville, FL 32611, USA

**Keywords:** fortification, iron, corn flour, program implementation, qualitative assessment

## Abstract

Background: In Mexico, the fortification of corn and wheat flours with iron, zinc, and folic acid and the restoration of B-vitamins is a mandatory program. However, the monitoring and evaluation (M&E) of this fortification process is not well understood. Thus, the purpose of the study was to understand the M&E of the food fortification program in Mexico, with an emphasis on technology research and development. Methods: Open-ended exploratory interviews were conducted with food technology representatives (n = 9), food science academic faculty (n = 1), president of a private tortilla-making federation (n = 1), and representatives of the federal monitoring agency (n = 2). Interviews were transcribed and themes were identified using the content analysis methodology. Inter-rater reliability was assessed by calculating an intraclass correlation coefficient (ICC) between the raters (n = 3). Results: A total of 49 codes were identified that resulted in three overarching themes, manufacturing/processing, monitoring logistics, and nutrition. Overall, there is a need for more robust internal and external M&E with Mexico’s fortification program to improve the manufacturing/processing of fortifying the tortillas, the monitoring of this fortification program, and the impact the fortified tortillas have on the nutritional status of the Mexican population. The overall ICC was 0.87. Conclusions: The present study can be used to gain insight into Mexico’s fortification program and to inform food fortification policymakers of best practices.

## 1. Introduction

**Figure 1:**
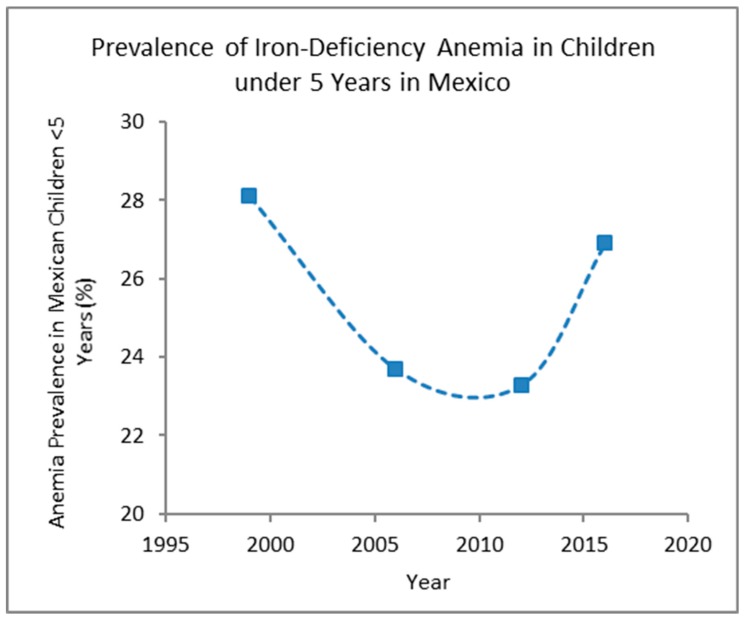
Prevalence of anemia in children under 5 years in Mexico (%). Data retrieved from Mexico’s national health and nutrition survey, ENSANUT (1999, 2006, 2012, and 2016).

Micronutrient malnutrition (hidden hunger) afflicts more than 2 billion people worldwide, especially women and children. Predominately those affected reside in low-resource regions and food insecure, low-income households [1,2]. The most prevalent micronutrient deficiencies are iron, iodine, and vitamin A [3,4,5,6]. Iron deficiency can lead to iron-deficiency anemia (IDA), resulting in an increased risk of developmental issues, infection, and death [2]. According to ENSANUT, 2012 (Mexico’s national health and nutrition survey), 23.3% of children who are <5 years of age and 17.9% of pregnant women who are between the ages of 12–49 years suffer from IDA [7,8]. Since the country’s first ENSANUT in 1999, the prevalence of IDA in children under 5 has declined slightly, but in recent years has risen (Figure 1) [9,10]. Moreover, the prevalence of IDA in Mexico remains a moderate public health significance according to the World Health Organization’s (WHO) scale for public health significance (≤4.9% no public health problem; 5.0–19.9% mild public health problem; 20.0–39.9% moderate public health problem; ≥40.0% severe public health problem) [11]. The WHO has responded by recommending food companies within Mexico to fortify their corn flours with iron to eradicate iron deficiency and its related complications [12].

Though IDA is a countrywide public health concern, Central Mexico suffers from high iron-deficiency and anemia rates. In Central Mexico, in 2012, it was estimated that 30.5% of women between the ages of 20–49 years suffered from iron deficiency [7], and in 2016, female preschoolers were significantly associated with higher odds of having anemia (odds ratio = 1.9, *p* < 0.05) [10]. Because of these concerns, efforts to combat iron deficiency have largely focused on this geographic region.

In Mexico, nixtamalization has been the process used since Mesoamerican times to treat corn with lime before grinding it to a dough (“nixtamal masa”, or just “nixtamal”) to make tortillas, which is the main staple food commodity [13,14]. Nixtamal masa can be flash dried to make a nixtamalized corn flour, which can also be utilized to make tortillas by rehydrating the flour to make dough. In addition to changing the flavor and texture of the tortilla, the nixtamalization process increases the nutritional value of corn tortillas such as dietary fiber, the bioavailability of amino acids and niacin, and calcium [13]. These tortillas are available for purchase at small, local tortillerías (neighborhood stores selling tortillas), supermarkets, and convenience stores, or made at home. Contrary to other parts of the world that consume corn flour, in Mexico, the nixtamalized corn flours are the ones mandated by the government to be fortified with iron, zinc and folic acid, and restored with the B-vitamins [15] due to this longstanding tradition.

The Mexican government’s Normas Oficiales Mexicanas (NOM) are technical regulations issued by various federal government agencies, which establish rules, specifications, attributes, guidelines, characteristics or requirements applicable to a product, process, installation, system, activity, service or method of production or operation. The NOM-247-SSA1-2008 details the production process of flours, including the fortification of flours [15]. This document outlines that a vitamin and mineral premix should be mixed into the flour and adequately dosed such that the final flour will contain zinc (40 mg/kg), iron (40 mg/kg), folic acid (2 mg/kg), niacin (35 mg/kg), riboflavin (3 mg/kg), and thiamine (5 mg/kg). This document is to be used by the governmental organization, Comisión Federal para la Protección contra Riesgos Sanitarios (COFEPRIS), a quality control agency responsible for the monitoring of compliance with these regulations, to ensure quality products for consumers [16]. However, the extent the nixtamalized corn and wheat flours on the market are adequately fortified is unknown, because there are no available reports on monitoring and evaluation (M&E), specifically for 30% of the corn flour market, which is comprised of small- to medium-scale operations [17,18]. There could be many reasons for the limited M&E of fortification, such as a lack of fortifying equipment [19], lack of flour producers’ fortification knowledge [20], or lack of proper monitoring equipment or tools (i.e., atomic emission spectroscopy in the case of iron) [21], which is often the case in low-resource settings [22].

To aid food companies in quantifying micronutrients within fortified food products, the WHO has published guidelines for developing analytical tools in low-resource settings, known as the ASSURED (Affordable, Sensitive, Specific, User-friendly, Rapid and Robust, Equipment-free, and Deliverable to end-users) criteria [23]. The ASSURED criteria can be applied to the development of inexpensive micronutrient analysis tools to support government food fortification policies in low-resource settings [22]. One example of such appropriate technology is the Nu3px smartphone iron sensor for fortified foods, which is a cost-effective and user-friendly alternative to its traditionally used gold standard counterpart, atomic emission spectroscopy [24]. Sensors can be used to monitor compliance and efficacy of food fortification programs, a critical component of fortification policies to fulfill the program’s stated objectives [1]. To have an adequate monitoring system, fortification experts recommend that internal monitoring of the fortification process (i.e., quality control by food processor) should be conducted daily and external monitoring (i.e., factory visits by external monitoring auditor) should be conducted once every 3–6 months, or every 1–4 months in the case of detected problems [1]. However, a recent narrative review of the literature showed that current methods for internal or external micronutrient detection in food lacks the affordability, precision, simplicity, robustness, and validation to comply with the WHO criteria for fortification programs [22].

Furthermore, a qualitative study was conducted to understand external monitoring of food fortification in Africa and Asia. Participants of this study included fortification experts (n = 11) and individuals (n = 18) from regulatory agencies and the food industry. The authors found that there was a lack of monitoring resources, unclear monitoring legislation, and variable perceived value of monitoring. Moreover, the authors were able to provide valuable and actionable recommendations to current and future fortification programs such as clear legislation, strong leadership, and compliance enforcement [19]. No known qualitative studies, though, have been conducted among stakeholders of fortification programs in Latin America to understand their M&E of fortified products within this context. The objective of the present work was to understand strengths and weaknesses of the M&E of Central Mexico’s nixtamalized corn and wheat flour fortification program and to provide recommendations for improvement within the Latin American context. Therefore, this exploratory qualitative study was conducted among fortification stakeholders in Central Mexico to understand the context of the nixtamalized corn and wheat flour fortification program and to identify strengths and weaknesses related to monitoring and evaluation (M&E).

## 2. Materials & Methods

### 2.1. Qualitative Research Approach

This qualitative study encompassed interviewing individuals in decision-making or influential positions from organizations (food industry, academia, government) in Mexico’s fortification program. The information discussed in this section will follow the Consolidated Criteria for Reporting Qualitative Research (COREQ) [25]. The stakeholders’ roles were considered direct (i.e., fortified food producers and processors) or indirect (i.e., subject matter experts and regulatory oversight). Stakeholder analysis was selected as an approach as it is a well-established method for identifying areas of growth and critical actors who can influence policy [26]. Stakeholders were identified through knowledge of the in-country nixtamalized corn flour producers and by in-country collaborators.

### 2.2. Phase 1: Participant Recruitment

Stakeholders were invited through an email invite and one follow-up reminder email occurred after 3 months through purposive sampling (n = 12) and snowball sampling (n = 1) in central Mexico (Estado de Mexico and Querétaro states) [27]. Central Mexico was chosen as the study’s location due to its proximity to many of the pertinent stakeholders of the fortification program (i.e., federal government and flour producing facilities). Stakeholders were identified as directors or managers of relevant companies and organizations, or experts in the field such as small-scale private nixtamalized corn flour companies (n = 6), private vitamin and mineral premix producers (n = 3), experts in the area from academia (n = 1), president of a private tortilla-making federation (n = 1), and federal government monitoring agency representatives (n = 2). Two academic experts declined participation for reasons of time and one nixtamalized corn flour company declined participation for privacy reasons. The researchers had no prior relationship with the study participants. All study protocols were granted ethical approval by the University of Illinois Institutional Review Board #18576.

### 2.3. Phase 2: In-Depth Interviews with Stakeholders

In-depth interviews (n = 6) were conducted in Mexico by two Spanish-speaking interviewers (doctoral researcher AWW and postdoctoral researcher AD, PhD) who were trained in conducting interviews, in August 2018. All of the interviews were conducted in-person in the Spanish language. Field notes were written as needed for non-verbal observations. Stakeholders signed a written consent form to participate in the interview, which included the goals of the study. Though varying slightly between stakeholder groups, the stakeholders were generally probed to discuss topics related to monitoring logistics, individual perceptions of fortification, and fortification procedures and processes. The interviews were expected to last between 30–45 min. Interviews were conducted in the stakeholders’ private offices or communal work meeting spaces, which was deemed as most convenient by the stakeholders. Only the researchers (n = 2) and the study participants were present for the interviews. Some of the organizations, tortillería (n = 1) and nixtamalized corn flour manufacturing plants (n = 2) also invited the interviewers for tours of their production facilities. The interviews were conducted in urban settings in the states of Querétaro and Estado de Mexico.

**Figure 2:**
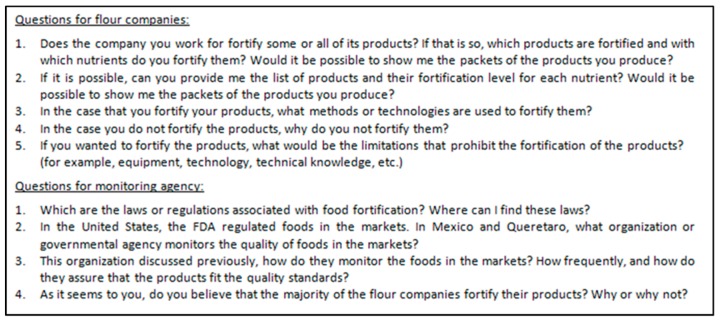
Interview questions translated to English for flour companies (n = 5) and monitoring agencies (n = 4).

Semi-structured, open-ended exploratory questions (designed to address the research question [28]) were used to obtain relevant context information about the fortification program (see Figure 2). The content of the interview questions was reviewed by two experts (doctorates of food science with experience in conducting research in Mexico) before deploying its use in Mexico and being revised accordingly. The guiding questions (n = 5) posed for stakeholders at a food company were different from questions (n = 4) posed to the government agency because of their different roles in the program. Responses were recorded via iPhone 8 Voice Memos application (audio recording device) and hand-written field notes to capture each response to the fullest. No compensation was provided to the participants. Transcripts were read by researchers (AWW and AD) and, as no new information was obtained, data saturation was considered reached.

### 2.4. Phase 3: Thematic Analysis

**Figure 3:**
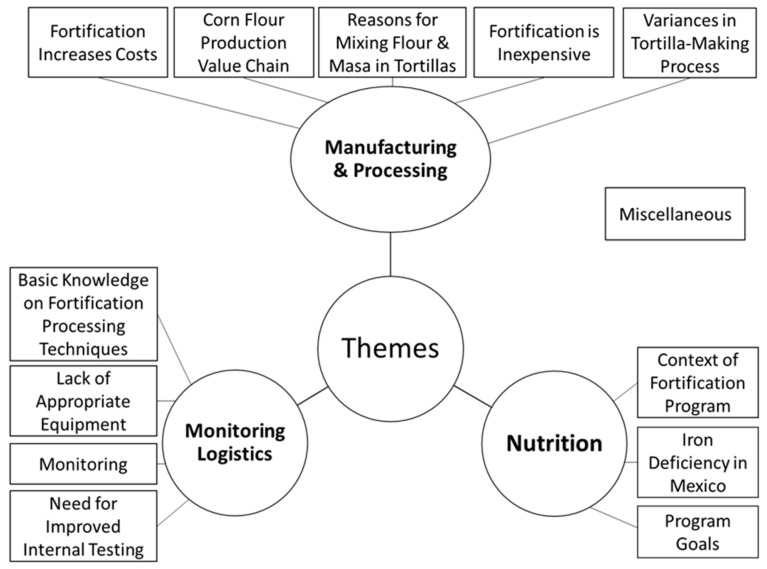
Thematic Map. Codes (n = 49) were grouped into concepts (n = 13, rectangular boxes in figure) and themes (n = 3, circles in figure).

The six phases of thematic analysis were undertaken for this study [29]. The entire thematic analysis process (coding and analysis) was conducted in Spanish. For Phase (1), interviews were transcribed verbatim by a Spanish speaker (AWW) and native Spanish speakers (AD and EB) into Microsoft Word. In Phase (2), three individual researchers (authors AWW, AD, and EB) independently read all the transcripts and identified main codes [26,28]. Codes were referred to as descriptors of a quotation [29]. Of the three lists of codes, 6 identical codes were independently identified by all three researchers, 10 identical codes were independently identified by two researchers, and 53 codes were independently identified by one researcher for a total of 69 codes. In Phase (3), researchers agreed on grouping similar codes into concepts (groups of similar codes). Researchers deliberated until one final agreed-upon list of codes (n = 51) grouped into concepts (n = 13) was generated. After consultation with qualitative researchers (authors JMA and JEA) familiar with the subject matter, two codes were deleted, and one concept was reworded. Thus, a final list of 49 codes and 13 concepts was identified. In Phase (4), researchers (authors AWW, AD, and EB) independently grouped similar concepts and their codes into themes (groups of similar concepts). The themes identified linkages between concepts. For Phase (5), all researchers deliberated until a final agreed-upon set of themes (n = 3) and definitions was generated, forming a thematic map (see Figure 3). Finally, for Phase (6), transcript documents, concepts, and codes were imported into Atlas.ti 8.1 software [30]. Researchers (authors AWW, AD, and EB) independently coded the transcripts using Atlas.ti. Frequencies were generated by Atlas.ti, and inter-rater reliability was calculated using intra-class coefficient (ICC) using SPSS v.24 statistical software [31]. Based on the ICC, reliability was considered poor (<0.5), moderate (0.5 to 0.75), good (0.75 to 0.9) or excellent (>0.9) [32]. After deliberation and agreement among all of the researchers, all six transcripts were coded. A final set of frequencies of codes were calculated on Atlas.ti. Based on agreement among the researchers, verbatim quotations from interviews were extracted to demonstrate the main themes. Researcher AWW translated the selected quotations from Spanish to English, and researcher AD verified their clarity. Participants did not provide feedback on the findings.

## 3. Results

The interviews ranged from 20 min to 2 h (mean length 50 min). From the data analysis of the interviews, three main themes were identified (and their subsequent concepts) and can be seen in Figure 3. Overall, ICC between the reviewers was 0.87, indicating good inter-rater reliability. Disagreements between reviewers tended to be prevalent in monitoring logistics codes. Thus, these codes were regrouped into concepts by the reviewers, and disagreements were discussed and deliberated until all three researchers (authors AWW, AD, and EB) agreed.

### 3.1. Monitoring Logistics

#### 3.1.1. External Monitoring Capacity

Government participants mentioned that the monitoring capacity has declined in recent years due to a lack of funding and laboratory infrastructure. The participants indicated that the program started out as a federally funded project, but it is no longer federally funded and has been the states’ responsibility for the last 5 years. Additionally, it was noted, by one participant, that each state in Mexico previously had its own authorized laboratory for samples; now, however, producers send samples across state lines, acquiring more fees and taxes.

#### 3.1.2. Frequency of External Regulatory Audits

Through the interviews, participants discussed that the lowered monitoring capacity (i.e., funding and laboratory infrastructure) has affected external monitoring checkup frequency. These participants discussed that the external monitoring can result in product recalls or manufacturing suspension. The participants indicated that the size of the food production will dictate the frequency of external monitoring visits, such that smaller production facilities will have fewer external monitoring checkups compared to larger companies. For example, a corn flour producer stated, “*These controls can be very varied. Maybe because of the size of the company, it [the checkups] has never touched us*”.

#### 3.1.3. Variation in External Regulatory Audit Processes

As the participants mentioned, government auditors review several aspects of the fortification process during their visits such as the sanitation conditions and the overall upkeep of these facilities, but these reviews are infrequent and inconsistent. They also mentioned that the fortification is monitored such as inputs and outputs of flour and premix, but the record keeping may not be accurate as alluded to by a government representative:

“*During the visits we review the addition of micronutrients. We have a NOM 251 that speaks of all Good Manufacturing Practices. We monitor everything that is the facilities, the process water, the drainage, the toilets, the ventilation… We also check the warehouse with special attention to the storage of flour that has been added and the premix, and so all this is documented. Then in the warehouse they must have a record of inputs and outputs*”.

#### 3.1.4. Internal Monitoring Capacity

As indicated by the participants, internal monitoring for small scale flour producers is accomplished using qualitative methods for iron determination in a yes/no binary system on a more frequent basis than the external validated analysis in authorized laboratories. As they mentioned, the qualitative method is utilized more often than the validated analysis due to its rapid and low-cost nature, but it is not always precise. When the researchers asked the participants if an alternative method was available to detect micronutrients as opposed to the qualitative method, the small-scale flour producers agreed an alternative technology would be better if it were cost-effective, faster, and more precise. As a vitamin and mineral premix producer stated, “*Customers are currently occupying the qualitative techniques* [to analyze micronutrient content] *for faster determinations. Because* [of cost] *they cannot send all the batches to analyze*”.

### 3.2. Manufacturing and Processing

#### Preparation of Tortillas

Participants described how nixtamalized corn flour is processed into tortillas, a main staple commodity for the Mexican diet. They indicated that the tortilla recipes vary from tortillería to tortillería. The recipes utilized by tortilleros (the people who make tortillas) can be made from fresh nixtamal masa (non-fortified), nixtamalized corn flour (fortified), or any ratio of both together. These differences are often based on local consumer preferences. Participants indicated that as a result of these variations, added micronutrient content in tortillas is likely to fluctuate. A research professor described how, “*In Mexico City, the tortillas are made of 70% nixtamal and 30% flour.* [Thus] *the fortification is diluted*”. Whereas a Tortilla-maker in Queretaro stated, “*We are using 95% pure corn* [non-fortified] *and 5% flour* [fortified] [to make tortillas]”. Which was reinstated by a corn flour producer, “*The problem we have… is that mixing nixtamal masa with nixtamalized corn flour makes the product* [the tortilla] *not 100%* [flour]. *So, although we fortify the flour correctly, the final consumer, is no longer acquiring* [the micronutrients] *in the tortilla*”.

Participants mentioned several reasons that tortilla producers use a blend of masa nixtamal and fortified nixtamalized corn flour. These reasons may include cost, texture, shelf-life, and flavor. As the president of a tortilla-making federation explained:

“*It* [the flour] *is added with softener, bleach, and some substance that serves to give strength or that does not break the tortilla when you make a taco. But, we are removing* [decreasing the use of] *those flours, to give a little texture, or softness to the tortilla. Why have we reduced the consumption of flour? First, we find it is more expensive to produce tortillas with flour than we do our own nixtamal… Without using a lot of corn* [fortified] *flour, we already have the softness, we have a very good texture, we have very good resistance of the tortilla to make a taco*”.

This was further verified by a research professor, “*[People eat the tortillas] for the taste. The fortified flours have a characteristic flavor. Here, we recognize the taste if you give someone a fortified flour tortilla. If you go to the north of the country, they are used to eating fortified flour. If you go to the center of the country and to the south of the country, they are not so used to eating fortified flours*”.

### 3.3. Nutrition

#### 3.3.1. Limited Evaluation of the Fortification Program’s Impact on Health

Since the implementation of the fortification program, it was noted by participants that the Mexican government has yet to conduct an efficacy trial to assess the program’s impact on the prevalence of iron deficiency or IDA amongst the population. Assessing efficacy is an important part of a successful program’s M&E process. Because of this limitation, it was noted that it is difficult to assess the impact the process of combining fortified nixtamalized corn flour and non-fortified masa nixtamal, in different quantities, has on health outcomes. Without a population-based approach to evaluate the fortification program’s efforts in reducing micronutrient deficiencies, it was noted that participants such as the government or the industry cannot be sure that the program is a cost-effective approach to improving public health as mentioned by a government representative:

“*We have not measured the contribution* [the fortification program] *on the health of the population. However, we believe that it is contributing* [to their health] *since the entire population practically consumes this, be it bread or tortilla*”.

#### 3.3.2. Limited Impact Divided by Geographic Location and Socioeconomic Status

Participants stated that the fortification program is currently perceived to be reaching limited segments of the Mexican population, such as those with a higher economic power, those situated in urban regions, and those in the north of the country. This is problematic for the program’s intended impact, which is to improve the micronutrient status on all populations throughout the country. Previous literature has documented higher iron deficiency prevalence in rural areas and the central and south of the country compared to other areas within the country [7,32], indicating a larger emphasis should be placed on these more vulnerable subpopulations as emphasized by a corn flour processor:

“*The fortification programs are for the population that has significant nutritional deficiencies. Our product is fortified because it is mandatory. But our product does not normally go to the population* [that needs it], *but to a higher purchasing sector*”.

#### 3.3.3. Diets of the Mexican Population

As the diets of the Mexican population evolve, the demand for tortillas is diminishing [33]. Thus, participants question the validity of fortifying this product as mentioned by a research professor:

“*We, as Mexicans, have changed our diet, we have stopped eating tortillas. We should see the consequences that this change in diet has had on these problems [micronutrient deficiencies] that are occurring*”.

## 4. Discussion

The purpose of the present work was to understand the context of the fortification program in Central Mexico and to identify strengths and weaknesses related to monitoring and evaluation (M&E) and to recommend strategies for improving its M&E. Three main themes were derived from the interviews through content analysis: 1. Monitoring logistics; 2. Manufacturing and processing; and 3. Nutrition. The main findings highlight the inconsistencies with program design and practice, such as the monitoring strategies, evaluation of the program impact, and actual consumption of nixtamalized tortillas made of flour. Stakeholder insights offered potential strategies for improving the program, such as increased internal monitoring technical capacity, increased fortification education for tortilleros, and raising awareness of the nutritional differences between nixtamalized tortillas made of fortified flour and tortillas made of masa nixtamal.

### 4.1. Monitoring Logistics Theme

The interviews revealed that in recent years, the access and use of authorized laboratories in Mexico for analyzing the micronutrient content of flour samples has declined. Furthermore, a lack of authorized laboratories ultimately increases the cost for nixtamalized fortified corn flour producers to monitor their products, as sending samples across state lines will accrue more taxes and fees for the analysis. For these reasons, the M&E component of the NOM-247 has decreased in volume and frequency of monitoring checks. The frequency of checks is correlated to the size of the company, as smaller companies will be audited less frequently. A parallel case can be seen in fortification programs in Africa and Asia, where similar budget restraints affect M&E rigor and frequency [19].

Literature shows that having a rigorous M&E component, in any mass mandatory fortification policy, is critical for understanding whether the program is reaching its nutritional goals within the context success [34]. Additionally, monitoring is required to identify problems such as non-compliance and implement corrective actions [1]. An ideal fortification program will have a small number of locations producing the fortified product, such that M&E resources can be consolidated [1]. Thus, in the case of fortified nixtamalized corn flours in Mexico, it presents an ideal case for a rigorous and successful M&E program, as one company dominates 70% of the corn flour sales, and the remaining 30% of the market is made up of a few, small flour companies, such as the ones interviewed within this study [17,18]. However, the perspectives of stakeholders outlined in this study demonstrate that gaps remain in the M&E capacity, in terms of the frequency and consistency of external checkups, and the use of imprecise internal qualitative methods (i.e., iron spot test).

The iron spot test is often recommended as a qualitative methodology for internal monitoring [35]. In this test, a mix of solution is added to the flour, then it will oxidize iron, if present, and thus, red spots will appear in the flour. The presence or absence of red spots indicate fortification in a binary system (yes/no), but it is not quantitative [36]. The iron spot test is estimated to cost $1 USD per assay plus laboratory staff costs [36]. Currently, the iron spot test is being utilized by the corn flour producers in Mexico. However, the stakeholders expressed an interest in a more precise alternative method, specifically one that is more cost-effective.

Furthermore, through the results of this study, the actions in case of non-compliance are not well understood. In the Compliance section (Section 12) of the NOM-247-SSA1-2008, it only states:

“*12. Compliance with the standard*

*12.1. The monitoring of the application of this rule corresponds to the Ministry of Health in the terms and applicable legal provisions*”.

The stakeholders mentioned product recalls or manufacturing suspensions as possible actions to take in the case of non-compliance, but there is no publicly documented protocol that supports a standardized procedure.

### 4.2. Manufacturing and Processing Theme

Fortified nixtamalized corn flour producers sell household-size packages of fortified nixtamalized corn flour that is sold in supermarkets or convenience stores. Additionally, it is known that these producers sell large, bulk quantities of fortified nixtamalized corn flour to tortillerías (neighborhood tortilla producing facilities). However, it is up to the tortilla producer’s discretion to produce their tortillas using all fortified nixtamalized corn flour, or whether they mix fortified nixtamalized corn flour into the traditionally used masa nixtamal. Prior literature indicates that in Mexico, tortillas are often made from a mix of fortified nixtamalized corn flours (dried) with traditional masa nixtamal (wet) [14,36]. The stakeholders identified several reasons for mixing traditional masa nixtamal with fortified nixtamalized corn flour such as enhanced texture, longer shelf-life, improved flavor, decreased cost, and flavor preferences. Production cost is lowered by using a mix of nixtamalized corn flour (as low as 5%) with their own masa nixtamal. It has been estimated that tortillerías that use a mix of nixtamalized corn flour and their own nixtamalized corn (to make the masa nixtamal themselves) can raise $559,312 MXN pesos (approximately $29,275 USD) per year in revenue more than their only masa nixtamal tortillería counterparts in the State of Mexico [37].

These variances in tortilla-making are dependent on regional consumer preferences. For instance, in Mexico City, the final blend may contain around 70% masa nixtamal and 30% fortified nixtamalized corn flour. However, in the state of Querétaro, an owner of several tortillerías uses only 5% fortified nixtamalized corn flour. This difference between flour ratios across regions highlights the uncertainty in the amount of fortified nixtamalized corn flour that is consumed by people who purchase their tortillas from tortillerías and not mainstream supermarkets. This variance undermines the potential for nutritional impact and dilutes the micronutrients reaching the consumers, as indicated by the stakeholders.

### 4.3. Nutrition Theme

IDA prevalence in Mexican children under 5 years of age decreased since the implementation of the mandatory flour fortification program to 23.3% from the years 1999 to 2006, but increased in 2016 to 26.9% (see Figure 1) [9,10]. The exact cause of the recent increase (2012–2016) is unknown, but experts believe it may be attributed to the nutritional transition occurring in Mexico, in which foods rich in iron are being replaced with foods rich in energy and poor in micronutrients [38]. While the Mexican government attributes the decrease in anemia prevalence (1999–2006) to the flour fortification program and other government nutrition initiatives, they also acknowledge a lack of impact evaluation of this program to confirm this hypothesis.

The assumption that Mexico’s corn flour fortification program is responsible for the reduction in countrywide anemia prevalence is contrary to other studies that demonstrate a lack of efficacy using corn flour fortified with electrolytic iron, an iron fortificant recommended by Mexico’s fortification policy, but with limited bioavailability [15]. A randomized controlled trial was conducted in Kenya to compare the efficacy of NaFeEDTA to electrolytic iron in 516 children between the ages of 3–8 years old who consumed iron-fortified whole maize flour five times a week. Concentrations of hemoglobin, plasma ferritin, and transferrin receptors after 5 months compared to placebo indicated that children consuming corn flours fortified with electrolytic iron showed no change in IDA, but there was a positive change (−89% IDA prevalence) when consuming corn flours fortified with 56 mg Fe/kg flour using NaFeEDTA [39].

Bioavailability of iron sources are heavily influenced by factors such as disease state and dietary patterns. Malaria, commonly present in developing countries, inhibits iron absorption due to increased inflammation. Electrolytic iron is highly susceptible to binding to phytates in cereals such as corn [39]. Thus, factors, such as bioavailability of iron source and consumption patterns, must be considered before making efficacy assumptions. Ideally, a randomized controlled trial of consumption of fortified nixtamalized corn flour in these tortillas among low-income individuals within Mexico could be conducted in order to substantiate any claims.

Additional limitations to identify the impact the fortification program has on nutritional outcomes of the population are related to socioeconomic status and geographic variation. It was identified, in the interviews, that the principal beneficiaries of this program would be people who live in the north of the country (where consuming 100% flour tortillas is more common), people in urban areas (who purchase 100% flour tortillas from convenience markets), or the upper class (who typically buy tortillas in a supermarket where the tortillas are made from flour). In the 2012 ENSANUT, 30.5% and 31.9% of women between the ages of 20–49 years were iron deficient in the central and south region of the country, respectively, while 22.8% of women of similar age in the north of the country were iron-deficient [7]. Similarly, the 2016 ENSANUT showed that adolescents (5–11 years) were more likely to have anemia in rural settings (15.7%) than urban (11.3%), and in the Southern regions (14.6%) than the northern (10.1%) [10]. In the 1999 and 2006 ENSANUTs, it was found that Mexican women were significantly more likely to have IDA belonging to a low socioeconomic level than their high or medium socioeconomic level counterparts [40]. Though these are nutrition indicators of the population at large and do not necessarily imply consumption or lack of consumption of fortified tortillas as causation, its findings correlate to the stakeholder perceptions that the iron fortification program’s principle beneficiaries (i.e., those receiving the benefits of a diet with increased iron) could be those situated in the north or urban areas, and/or those with a higher socioeconomic status.

### 4.4. Recommendations

Comprehensive evaluation of fortification programs requires adequate and constant M&E [34]. Based on the uncertainties surrounding the impact and utilization of the flours for reducing IDA within Mexico, the authors recommend that a more rigorous M&E be implemented in Mexico’s fortification program. This includes a population-based evaluation that can assess the reduction of anemia in response to consuming tortillas made with fortified nixtamalized corn flours alone or in a mix with masa nixtamal. The evaluation should focus on all sectors of society who may benefit from this program, including rural, urban, and other geographical zones, and different socioeconomic strata [1]. The authors recommend a more thorough and frequent internal monitoring system (such as implementing Nu3px, an alternative ASSURED-designed iron quantification tool previously mentioned) to ensure quality fortified products [24]. Finally, the NOM could be modified to include a standardized protocol for actions to take in the case of non-compliance.

The quality of fortified products can also be enhanced through educating tortilleros on the importance of using fortified flours in tortillas for nutritional deficiencies. One way of doing this is to conduct continuous trainings to improve processes in the form of in-person seminars. Park et al. found that one in-person 30-min training and a 2-week follow-up training amongst low-technical skilled food handlers, increased their knowledge of food safety practices by 30% [41]. Consumers should be empowered to understand the difference between tortillas made from masa nixtamal and fortified nixtamalized corn flour in order to best decide the nutrition for their family. A government initiative can be implemented that uses both education and marketing strategies to educate tortilleros on the importance of using fortified nixtamalized corn flours in their tortillas and to raise awareness for Mexican consumers about the significance of consuming fortified foods in the diet. Rothschild describes how self-interests (i.e., production costs for a tortillero) can influence whether or not an individual is prone to behavior change, and how to navigate individual self-interests when designing an effective educational and marketing strategy [42]. In both cases, the educational initiatives should be designed such that each stakeholder understands the benefit that they have to gain by producing or consuming fortified products in a communication medium that meets the target audience at their present cognitive abilities [1]. An example of a successful large-scale program to improve nutrition in Mexico can be seen by the PROGRESA program in which a combination of education, health care, and cash transfers influenced families’ decisions to consume nutrient supplements [43]. Efforts to introduce a fortification educational and awareness initiative may be drawn from the education component of PROGRESA.

One limitation of the present study is that an interview with the largest corn flour producer (70% of the market) was not able to be conducted for privacy and access reasons. Furthermore, interviews were geographically limited within the states of Estado de Mexico and Querétaro and do not represent the perspectives of stakeholders from every part of the country. The results presented within this research are specific to the context of Mexico’s program and should not be extrapolated to other contexts without conducting similar interviews with other stakeholders. Finally, the interviews were limited to those people available and willing to participate, but did not include NGOs, other government offices beyond the regulatory agency, or beneficiaries (consumers).

In conclusion, the present study was a qualitative evaluation of Mexico’s flour fortification program using interviews with stakeholders from various perspectives: academic, public sector, and private sector. It was found that there are a reduced number of laboratories capable of providing accurate and reliable data to support M&E activities of the program. Nixtamalized corn flour producers are interested in inexpensive, fast, and precise alternatives to support their quality control processes. The fortification efforts in central Mexico may be diluted by mixing masa nixtamal with fortified nixtamalized corn flour for motives of texture, shelf-life, cost, and flavor. Finally, the principal beneficiaries of the program may be those found in urban areas, in parts of the country where consuming tortillas made from 100% flour is more common, and/or with a higher socioeconomic power. Recommendations to improve the program include the implementation of simpler technologies for frequent internal monitoring of the fortification process and to have an external entity evaluate the impact of the program on the anemia prevalence in the country. Future studies should include a more expansive qualitative assessment of the program, including interviews with consumers, as well as the larger production companies.

## Abbreviations

ASSURED	(Affordable, Sensitive, Specific, User-friendly, Rapid and robust, Equipment-free, Deliverable)
COFEPRIS	(Mexico’s Federal Commission for Protection against Health Risks)
ENSANUT	(Mexico’s National Health and Nutrition Survey)
ICC	(Intraclass Correlation Coefficient)
NOM	(Norma Oficial Mexicana)
WHO	(World Health Organization)
IDA	(Iron-Deficiency Anemia)
